# Quantifying *in situ* phenotypic variability in the hydraulic properties of four tree species across their distribution range in Europe

**DOI:** 10.1371/journal.pone.0196075

**Published:** 2018-05-01

**Authors:** N. González-Muñoz, F. Sterck, J. M. Torres-Ruiz, G. Petit, H. Cochard, G. von Arx, A. Lintunen, M. C. Caldeira, G. Capdeville, P. Copini, R. Gebauer, L. Grönlund, T. Hölttä, R. Lobo-do-Vale, M. Peltoniemi, A. Stritih, J. Urban, S. Delzon

**Affiliations:** 1 BIOGECO, INRA, Université de Bordeaux, Pessac, France; 2 Forest Ecology and Forest Management Group, Wageningen University & Research, Wageningen, The Netherlands; 3 Università degli Studi di Padova, Dep. TeSAF, Legnaro (PD), Italy; 4 PIAF, INRA, Université Clermont-Auvergne, Clermont-Ferrand, France; 5 Swiss Federal Institute for Forest, Snow and Landscape Research WSL, Birmensdorf, Switzerland; 6 Climatic Change and Climate Impacts, Institute for Environmental Sciences, Geneva, Switzerland; 7 Department of Forest Sciences, University of Helsinki, Helsinki, Finland; 8 Forest Research Centre, School of Agriculture, University of Lisbon, Tapada da Ajuda, Lisboa, Portugal; 9 Wageningen Environmental Research (Alterra), Wageningen, The Netherlands; 10 Department of Forest Botany, Dendrology and Geobiocoenology, Mendel University, Zemědělská 3, Brno, Czech Republic; 11 Natural Resources Institute Finland (Luke), Latokartanonkaari 9, Helsinki, Finland; 12 Swiss Federal Institute of Technology ETH, Planning of Landscape and Urban Systems, Zurich, Switzerland; Austrian Federal Research Centre for Forests BFW, AUSTRIA

## Abstract

Many studies have reported that hydraulic properties vary considerably between tree species, but little is known about their intraspecific variation and, therefore, their capacity to adapt to a warmer and drier climate. Here, we quantify phenotypic divergence and clinal variation for embolism resistance, hydraulic conductivity and branch growth, in four tree species, two angiosperms (*Betula pendula*, *Populus tremula*) and two conifers (*Picea abies*, *Pinus sylvestris*), across their latitudinal distribution in Europe. Growth and hydraulic efficiency varied widely within species and between populations. The variability of embolism resistance was in general weaker than that of growth and hydraulic efficiency, and very low for all species but *Populus tremula*. In addition, no and weak support for a safety vs. efficiency trade-off was observed for the angiosperm and conifer species, respectively. The limited variability of embolism resistance observed here for all species except *Populus tremula*, suggests that forest populations will unlikely be able to adapt hydraulically to drier conditions through the evolution of embolism resistance.

## Introduction

Massive forest mortality events due to drought stress and rising temperatures have been observed at the global and regional scales [1–5]. Considering that climate change models predict further increases in mean temperature and in the frequency and severity of extreme drought events [6], more negative impacts on tree survival are expected [7]. In this context, assessments of the properties associated with drought resistance in trees, and of the capacity of species to deal with environmental changes, may help us to anticipate the impact of climate change on forest tree species.

Hydraulic failure due to xylem embolism is now considered one of the main causes of drought-induced tree mortality [8–10]. When soil water potential drops due to water shortage, the tension of the xylem water column increases, promoting the formation of embolisms that reduce the hydraulic functioning of the plant [11,12]. In cases of prolonged drought, soil water potential continues to fall, triggering the spread of embolisms throughout the xylem conduit network, leading to the hydraulic dysfunction of the plant vascular system and, finally, to lethal damage to the plant [13,14,15]. Therefore, determining the resistance to embolism of the species is crucial for evaluating the consequences that the expected increase in drought event frequency can have on a given population, forest or biome. *P*_50_ is the xylem pressure at which 50% of conductivity is lost due to embolism formation, and it is widely used to assess plant hydraulic safety to embolism. Xylem-specific hydraulic conductivity (*K*_S_), i.e. the rate of water transport through a given area of sapwood per unit pressure difference and per unit length, is commonly used to assess hydraulic efficiency [12]. Across species, literature shows a weak correlation between hydraulic safety and hydraulic efficiency, but the absence of species displaying both high hydraulic efficiency and safety suggests a possible safety-efficiency trade-off [16]. In conifers, *P*_50_ and *K*_s_ are only weakly correlated, as embolism resistance is driven mostly by the torus-aperture overlap in pit pairs [17–19], whereas xylem hydraulic efficiency is not influenced by this pit trait. By contrast, in angiosperms, both *P*_50_ and *K*_s_ are associated with pit membrane structure [20–22] and thickness [23], as well as with the perforation structure [24,25].

Differences in resistance to embolism, i.e. in *P*_50_, across species have been widely reported [17,19,26,27,28]. However, less attention has been paid to within-species phenotypic variation in this hydraulic property. Phenotypic variability results from a combination of genetic variation (differences in genotype among different individuals within the population and between populations) and phenotypic plasticity (genotype property to render different phenotypes in different environments [29]), and defines the capacity of populations to succeed under changing environmental conditions [30,31]. Low levels of phenotypic variability across large spatial scales may indicate a low potential of species to adapt to ongoing climate change. Contrary to other plant functional properties (see for instance [32] for leaf phenology, [33] for leaf functional traits), previous works on hydraulic properties show that phenotypic differences within species are by far lower than those found across species [34–38], and these differences are even smaller in gymnosperms than in angiosperms [39]. For instance, Lamy et al. 2011, 2014 found neither phenotypic variability *in situ* and nor genetic differentiation between maritime pine populations and suggested uniform selection rather than genetic drift, for *P*_50_. However, whether the phenotypic variation of hydraulic properties varies across species distribution ranges remains largely unexplored. Furthermore, studies assessing the extent to which phenotypic variability in hydraulic properties is lower than that of other key species traits over large scales are also lacking.

The main aim of this study was to evaluate phenotypic variability in the functional hydraulic safety and efficiency (*P*_50_ and *K*_S_) of four European tree species (two conifers and two angiosperms) along a latitudinal gradient covering most of their distribution range. We assessed the capacity of the species to adapt to changing environmental conditions by exploring the links between hydraulic properties and latitude and climate. We also evaluated the phenotypic variability of branch growth in trees from the same populations to assess the extent to which hydraulic properties were conserved relative to other key traits. Finally, we assessed the safety-efficiency trade-off at intraspecific level. We hypothesized 1) a weaker phenotypic variation for hydraulic safety than for hydraulic efficiency and branch growth, given the highly conserved evolutionary nature of *P*_*50*_ [37,38]; 2) a phenotypic cline -a gradual change of a phenotypic character in a species over a geographical area- in both hydraulic safety and efficiency and 3) a weak safety-efficiency trade-off within species. This study provides for the first time a multi-species assessment of inter and intra-specific phenotypic variability in functional hydraulic properties along a large latitudinal gradient. Our results will help to characterize the adaptive capacities of European forests, which will have to face drier and warmer climatic conditions in the future.

## Materials and methods

### Study species and populations

We focused on four widely distributed European species, with different water-transport structures, from diffuse porous with scalariform perforation plates (*Betula pendula* Roth) or with simple perforation plates (*Populus tremula* L.) to softwood (two tracheid-bearing species, *Picea abies* (L.) Karst and *Pinus sylvestris* L.). For each species, four to six populations were selected across their distribution range (see the distribution range of the species and the location of the populations in S1 Fig). The mean annual temperature and total annual rainfall across selected populations ranged from -1.8 to 9.5°C and 538 to 1739 mm, respectively (S1 Table). We also selected two different sites a few kilometres apart, for each population.

### Climatic data

Data for mean annual temperature (MAT) and total annual precipitation (MAP) were obtained from WorldClim original 30-s data (http://www.worldclim.org/bioclim) [40] downscaled to 100-m resolution based on a high-resolution digital elevation model (DEM) and moving window regression technique [41] for all but the Italian (IT) and Swiss populations (SW-LOE and SW-PFY). The MAP data for the IT (Italy), SW-LOE (Switzerland-Loetschental) and SW-PFY (Switzerland- Pfynwald) populations were obtained from nearby weather stations at San Vito di Cadore (Centre for Alpine Environment Studies) and Sierre (www.meteoswiss.ch), respectively, due to considerable variations in topography. The aridity index (AI) was calculated as MAP/PET (total annual precipitation/annual potential evapotranspiration). PET was extracted from the Global Aridity and PET Database (http://www.cgiar-csi.org). We averaged the mean temperatures (T_Sum) or aridity indices (AI_Sum) of June, July and August to obtain mean values for the summer (see S1 Table for the climatic conditions of the populations studied).

### Xylem vulnerability to embolism

We collected branches from five to 11 healthy mature trees per population in the early morning during the wet season (spring 2015). One or two branches with three to five functional rings were sampled at mid-crown and south oriented. Samples had a standard length of 45 cm. Transpiration losses were prevented by removing the leaves or needles immediately after sampling and wrapping the branches in moist paper to keep them humid and cool (3°C) until the measurement of embolism resistance (within three weeks of sampling). The bark was removed from conifer branches to prevent resin to fill the cavitron reservoirs (see below, [17]), and all branches were recut with a razor blade, under water, to a standard length of 0.27 m. For each angiosperm species, 10 samples per species were used to test the open vessel artefact [42] by injecting air at 2 bars at one end and no open vessels were detected for any of them.

Vulnerability to drought-induced embolism was determined at the Caviplace (University of Bordeaux, Talence, France; http://sylvain-delzon.com/caviplace) and INRA-Clermont-Ferrand facilities, with the Cavitron technique [43,44]. Samples were infiltrated with a reference ionic solution of 10 mm 25 KCl and 1 mm CaCl_2_ in deionized ultrapure water. Centrifugal force was used to generate negative pressure into the xylem and induce cavitation. This method allows to measure xylem conductance under negative pressure using the custom software Cavisoft 4.0 (Univ. Bordeaux, Pessac, France). Initially, the maximum conductance of stem (*K*_max_, in m^2^MPa^-1^s^-1^) was calculated under low xylem pressures. The percentage loss of conductance (PLC) of the stems was calculated at different xylem pressures (P_i_) from -0.8 to -5 MPa with the following equation:

$$PLC=100\;\left(1-\frac{K}{Kmax}\right)$$

We obtained one vulnerability curve per tree by measuring one or two of the collected branches. These vulnerability curves show the percentage loss of xylem conductance as a function of xylem pressure [17]. For each branch, the relationship between PLC and xylem water pressure was fitted with the following sigmoidal equation [45]:

$$PLC=\frac{100}{\left(1+exp\;(\frac{S}{25}*\left(P_{i}-P_{50}\right))\right)}$$

where *P*_50_ (MPa) is the xylem pressure inducing a 50% loss of conductivity and *S* (% MPa^-1^) is the slope of the vulnerability curve at the inflection point. All sigmoidal functions were significant and fitted with the NLIN procedure in SAS (version 9.4 SAS Institute, Cary, NC, USA). The xylem-specific hydraulic conductivity (*K*_s_, kg m^1^MPa^-1^ s^-1^) was calculated by dividing the hydraulic conductivity measured at low speed by the sapwood area of the sample.

### Branch growth measurements

We also collected one branch per tree from three to five trees per population and site. We selected straight branches and did not keep any sample with reaction wood for our measurements. The allometric relationship between branch radius (mm) and xylem age (number of years) was used as a surrogate for tree growth, as radial branch growth and tree growth patterns are highly correlated [46]. The branch surface area and the number of tree rings were systematically measured at 70 cm from the branch apex.

### Statistical analyses

We assessed the phenotypic variability of functional hydraulic properties (*P*_50_ and *K*_s_) and branch growth (branch radius/xylem age) in each species, by testing the effect of population and site with nested ANOVAs, in which the population and the site nested in population were considered factors. If statistically significant differences were observed, post-hoc Tukey tests were conducted for multiple comparisons between populations. Before running the ANOVAs, we checked that the data satisfied the assumptions of normality and homoscedasticity. As vessel size can rapidly increase with branch size during early years of tree growth, and then may have a potential effect on hydraulic conductivity, we tested any potential correlation between K_s_ and branch diameter. We also calculated the inter-population and intraspecific coefficients of variation (% CV_inter_ and CV_sp_, respectively). For each species, Spearman´s or Pearson´s correlation coefficients (depending on the linearity condition) were calculated between the averaged by site *P*_50_, *K*_s_ and branch growth and the latitude and the five climatic variables mentioned above. Finally, we also checked for intraspecific safety-efficiency trade-offs, with Spearman´s or Pearson´s correlation tests.

Statistical analyses were performed with the R project for statistical computing (R Development Core Team, 2016) [47].

## Results

### Phenotypic variability across species distribution ranges

**Fig 1:**
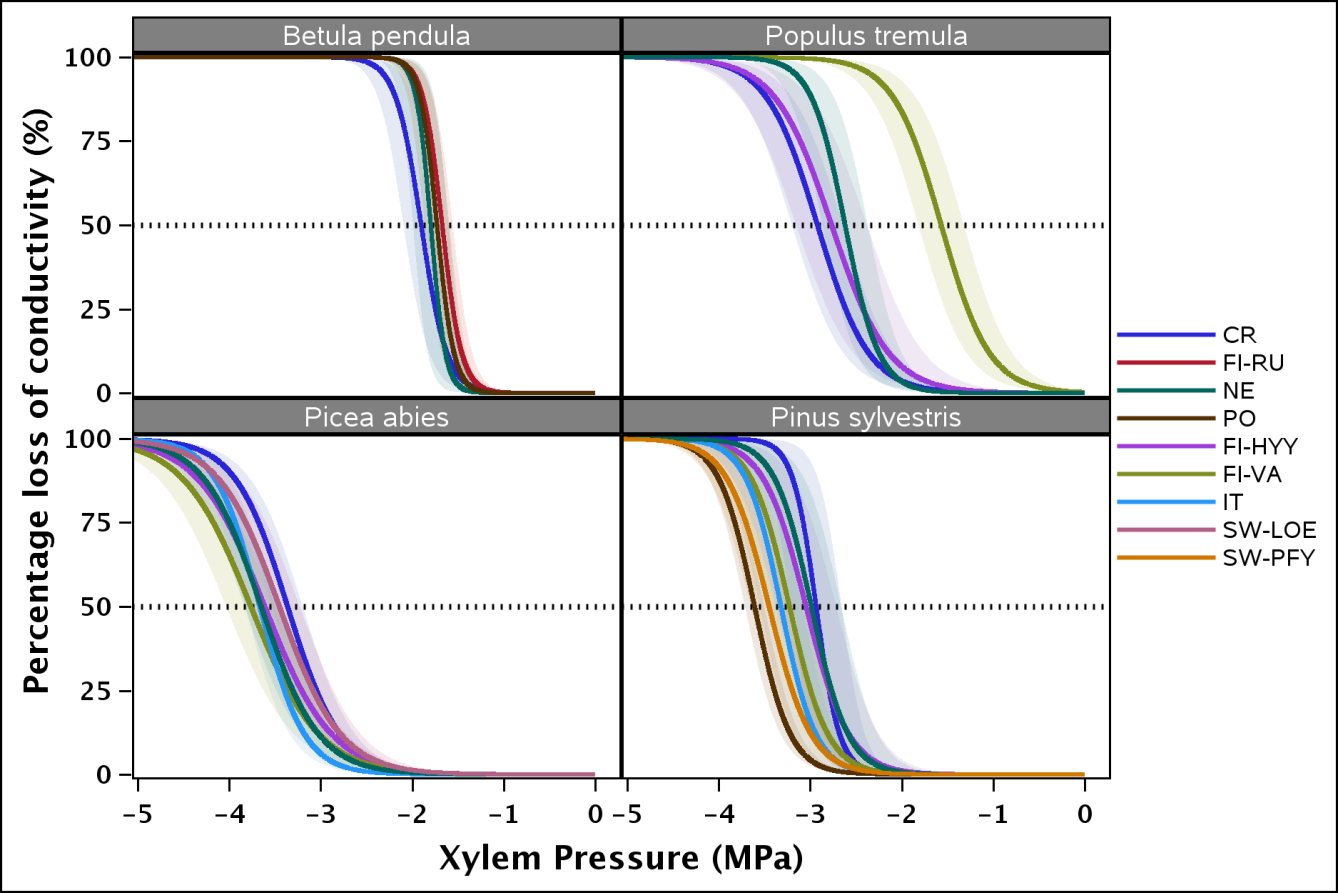
Xylem vulnerability curves for each population of the four species studied (*Betula pendula*, *Populus tremula*, *Picea abies* and *Pinus sylvestris*). The shaded band represents the standard deviation. CR: Czech Republic; Fi-RU: Finland-Ruotsinkylä; NE: The Netherlands; PO: Portugal; Fi-HYY: Finland-Hyytiälä; Fi-VA: Finland-Värriö; IT: Italy; SW-LOE: Switzerland-Loetschental; SW-PFY: Switzerland- Pfynwald.

Xylem vulnerability curves followed a sigmoid function in all species (Fig 1, S2 Fig), showing the lack of an open vessel artefact and the accuracy of the results obtained here. *Betula pendula* and *Picea abies* showed, respectively, the lowest and highest resistance to embolism of the four species evaluated. The mean *P*_50_ ± SE (MPa) was -1.78 ± 0.02 for *Betula pendula*, -2.45 ± 0.08 for *Populus tremula*, -3.16 ± 0.03 for *Pinus sylvestris*, and -3.58 ± 0.02 for *Picea abies* (Fig 1).

**Fig 2:**
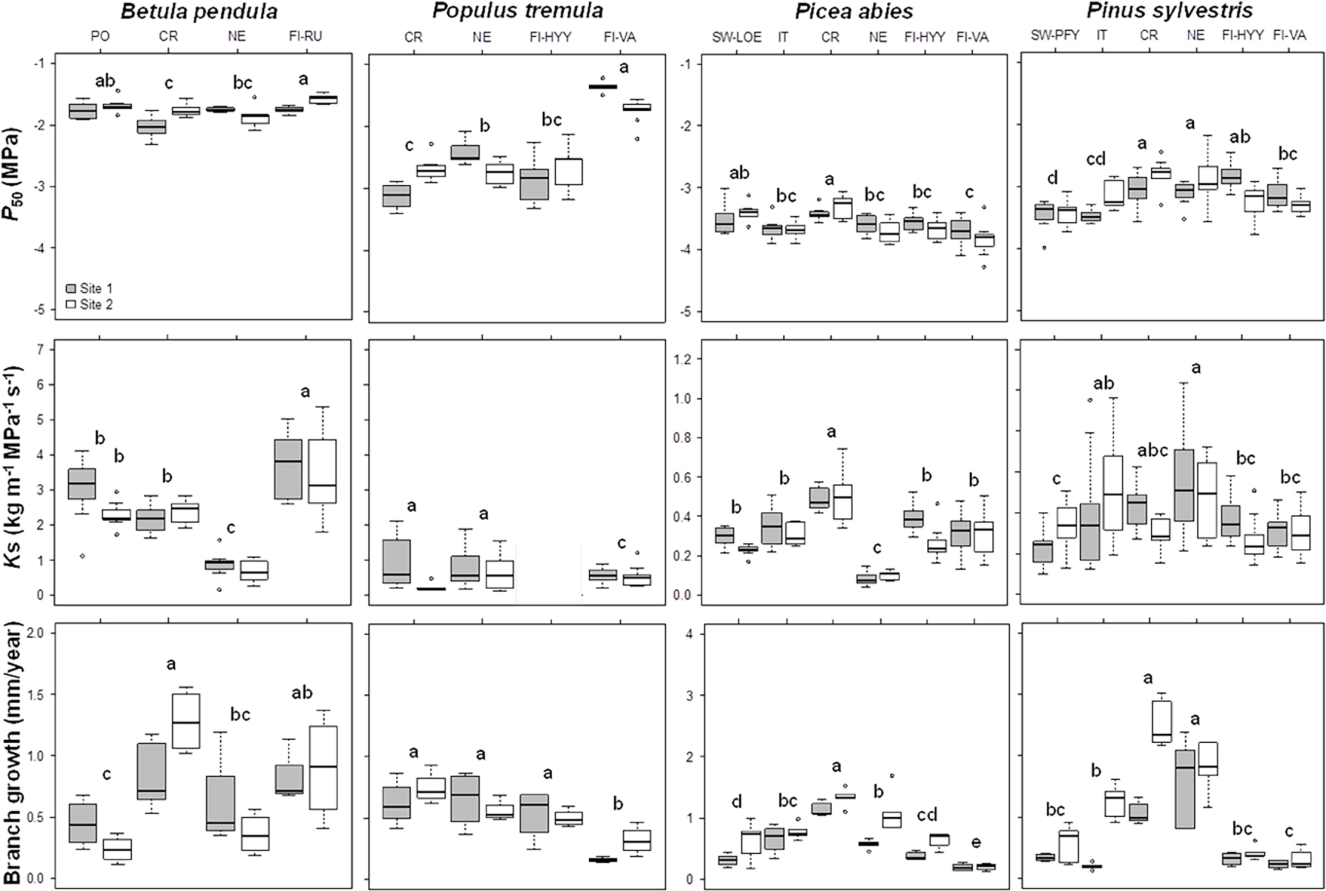
Mean *P*_*50*_ (MPa), xylem-specific hydraulic conductivity (*K*_*s*_, kg m^-1^ MPa^-1^ s^-1^) and branch growth (mm/year) per species, population and site. The two sites are represented in different colours (white and grey). The bars represent the nominal range of data variation, with the upper and lower ends showing the upper quartile plus 1.5 times the interquartile range and the lower quartile minus 1.5 times the interquartile range, respectively. Values beyond these limits are plotted as circles. CR: Czech Republic; PO: Portugal; NE: The Netherlands; Fi-RU: Finland-Ruotsinkylä; Fi-VA: Finland-Värriö; Fi-HYY: Finland-Hyytiälä; IT: Italy; SW-LOW: Switzerland-Loetschental; SW-PFY: Switzerland- Pfywald. Different letters indicate statistically significant differences between populations.

Differences in *P*_50_ between populations were observed for all species, whereas the site (nested in population) had an effect on *P*_50_ in all species but *Picea abies* (Table 1, Fig 2). The CV in *P*_50_ was low for all species other than *Populus tremula*. The variability in *P*_50_ of *Betula pendula*, *Picea abies* and *Pinus sylvestris* ranged from 4.15 (CV_inter_ of *Picea abies*) to 10.23% (CV_sp_ of *Pinus sylvestris*), whereas that of *Populus tremula* ranged from 24.82 (CV_inter_) to 25.07% (CV_sp_) (Table 2). The high variability observed for *Populus tremula* was mostly due to the population of Finland-Värriö (FI-VA), which had the least negative *P*_*50*_ values of any of the populations studied (Fig 2).

**Table 1:** Effects of population and site on *P*_*50*_ (MPa), xylem-specific hydraulic conductivity (*K*_*s*_, kg m^-1^ MPa^-1^ s^-1^) and branch growth (BG, estimated as branch radius/xylem age (mm/year)) of study species, according to nested ANOVAs. The F, p-values and degrees of freedom are shown. Pop: population.

			Angiosperms					Conifers						
			Betula pendula		Populus tremula			Picea abies					Pinus syvestris	
		df	F	p	df	F	p	df	F	p		df	F	p
	Population	3	**9.978**	**<0.001**	3	**124.885**	**<0.001**	5	**7.971**	**<0.001**		5	**15.284**	**<0.001**
P_50_	Site (Pop)	4	**7.894**	**<0.001**	4	**9.905**	**<0.001**	6	1.042	0.405		6	**6.505**	**<0.001**
	Population	3	**34.949**	**<0.001**	2	0.249	0.781	5	**31.381**	**<0.001**		5	**4.810**	**<0.001**
K_s_	Site (Pop)	4	1.022	0.405	3	2.809	0.051	6	1.743	0.123		6	2.019	0.070
	Population	3	**10.957**	**<0.001**	3	**12.999**	**<0.001**	5	**37.755**	**<0.001**		5	**50.702**	**<0.001**
BG	Site (Pop)	4	2.140	0.107	4	1.021	0.416	6	**5.539**	**<0.001**		6	**14.674**	**<0.001**

**Table 2:** Intraspecific (CV_sp_) and inter-population (CV_inter_) coefficient of variability (%) for the xylem pressure inducing a 50% loss of conductance (*P*_50_, MPa), xylem-specific hydraulic conductivity (*K*_*s*_, kg m^-1^ MPa^-1^ s^-1^) and branch growth (BG, estimated as branch radius/xylem age (mm/year)) for each study species.

		CV_sp_			CV_inter_	
Species	*P*_50_	*K*_s_	BG	*P*_50_	*K*_s_	BG
*Betula pendula*	9.67	53.47	58.94	5.56	50.28	47.05
*Populus tremula*	25.07	80.04	43.29	24.82	14.16	37.66
*Picea abies*	6.57	49.07	57.49	4.15	42.68	53.48
*Pinus sylvestris*	10.23	48.41	85.81	6.45	23.80	76.67

Variability levels were much higher for *K*_s_ than for *P*_50_ (Table 2, Fig 2). *K*_s_ differed significantly between populations, for all species other than *Populus tremula* (Table 2, Fig 2). For this species, we could not obtain absolute values of *K*_*s*_ for the FI-HYY population, due to software recording issues. No differences between sites were observed for *K*_s_ (Table 1). *Populus tremula* had the smallest CV_inter_ of the four species studied (14.16%), but the largest CV_sp_ (80.04%, Table 2). *Betula pendula* had the largest CV_inter_, with a mean difference of up to 2.8 kg m^-1^ MPa^-1^ s^-1^ between the populations located at the extreme ends of its latitudinal distribution range (Table 2, Fig 2). When significant, the correlations between *K*_*s*_ and branch diameter were weak (*Betula pendula* rho = -0.348, p = 0.008; *Pinus sylvestris* rho = 0.267, p = 0.005; *Populus tremula* rho = 0.090, p = 0.542; *Picea abies* rho = -0.210, p = 0.055).

Finally, branch growth differed between populations for all species, whereas site (nested in population) had a significant effect on branch growth only for conifers (Table 1, Fig 2). The phenotypic variability of branch growth was greater than that of *P*_50_ (Table 2, Fig 2). Furthermore, the phenotypic variability of branch growth was greater than that of *K*_s_ in most cases (Table 2, Fig 2). *Pinus sylvestris* had the largest CV_sp_ and CV_inter_ in branch growth (85.81 and 76.67%, respectively), whereas these two coefficients were the lowest in *Populus tremula* (43.29 and 37.66%, respectively) (Table 2).

### Phenotypic clines with climate and latitudinal gradients

**Fig 3:**
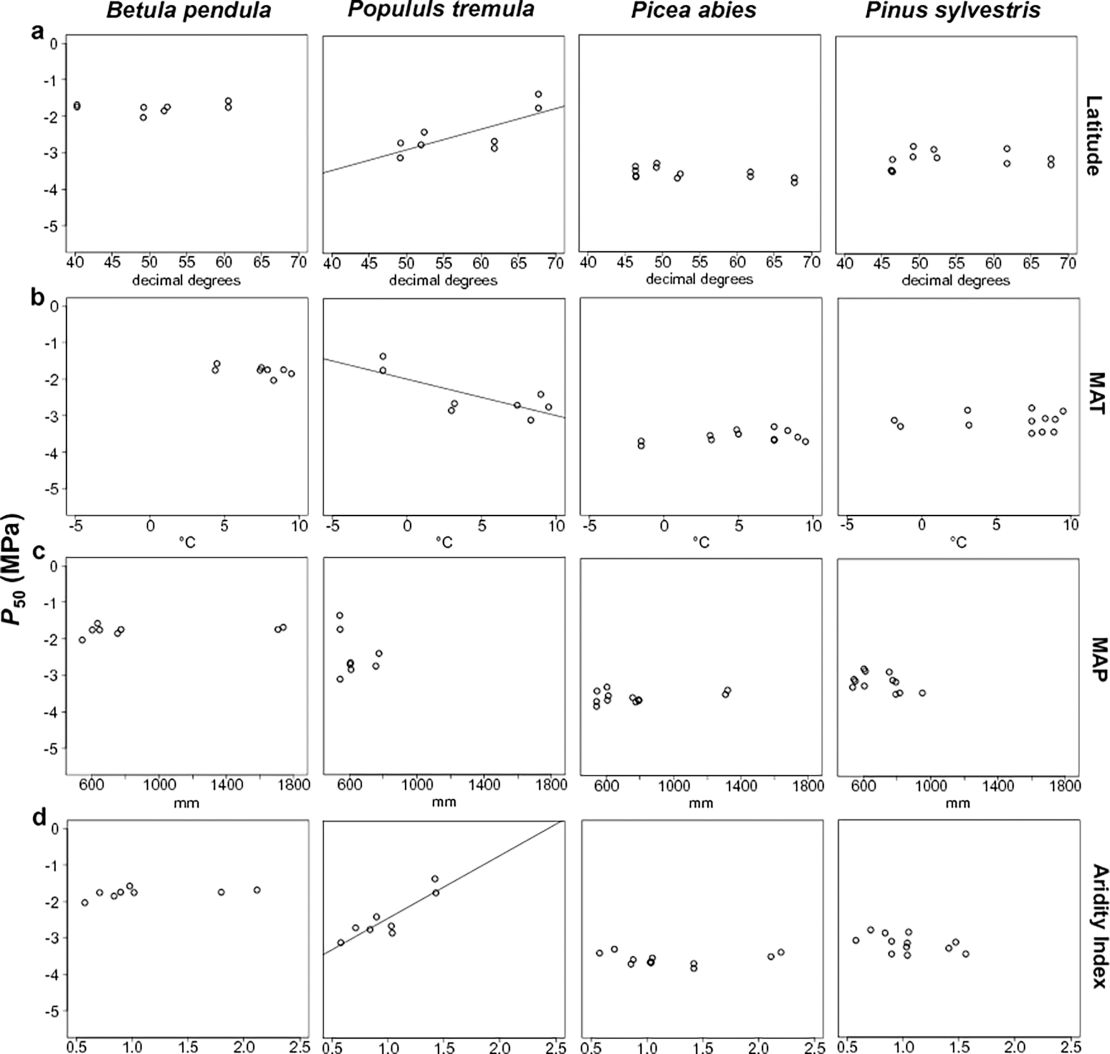
Mean *P*_*50*_ (MPa) per population plotted against latitude (3.a, decimal degrees) and the climatic variables for each sampled population and site: 3.b. mean annual temperature (MAT, °C); 3.c. total annual precipitation; (MAP, mm); 3.d. AI: aridity index (MAP/PET or potential evapotranspiration).

*Populus tremula* presented strong significant clines in *P*_50_, as five out of the six climatic variables studied here showed significant correlations with *P*_50_ (Table 3, Fig 3). *P*_50_ values for this species were positively correlated with latitude and aridity index (AI and AI_Sum), but negatively correlated with MAT and T_sum (Table 3, Fig 3). There was also a statistically significant negative correlation between *P*_50_ and T_Sum in *Betula pendula* (Table 3). By contrast, no significant clines in *P*_50_ were observed for conifers (Table 3, Fig 3).

**Table 3:** Correlation coefficients (Pearson or Spearman) and p-values for the relationships between the mean xylem pressure inducing a 50% loss of conductance (*P*_50_, MPa), xylem-specific hydraulic conductivity (*K*_s_, kg m^-1^ MPa^-1^ s^-1^) and branch growth (BG, estimated as branch radius/xylem age (mm/year)) and the climatic variables for each sampling site.

		*Betula pendula*		*Populus tremula*		*Picea abies*		*Pinus sylvestris*	
		Cor.	p	Cor.	p	Cor.	p	Cor.	p
P_50_	Latitude	0.167	0.692	**0.750**	**0.032**	-0.568	0.054	0.193	0.547
	MAT	-0.547	0.161	**-0.770**	**0.025**	0.420	0.174	0.056	0.862
	MAP	0.539	0.168	-0.214	0.610	0.288	0.364	-0.466	0.127
	AI	0.460	0.251	**0.886**	**0.003**	-0.112	0.728	-0.462	0.130
	T_Sum	**-0.793**	**0.019**	**-0.909**	**0.002**	0.341	0.278	-0.120	0.711
	AI_Sum	-0.289	0.487	**0.934**	**0.001**	0.098	0.761	-0.578	0.049
K_s_	Latitude	0.228	0.586	-0.166	0.753	0.001	1.000	-0.122	0.704
	MAT	**-0.886**	**0.003**	0.308	0.553	-0.147	0.649	0.408	0.187
	MAP	-0.119	0.779	0.086	0.872	-0.414	0.181	0.276	0.384
	AI	0.231	0.582	-0.206	0.695	-0.239	0.455	-0.279	0.379
	T_Sum	-0.428	0.290	0.292	0.575	0.082	0.799	0.225	0.481
	AI_Sum	-0.180	0.670	-0.439	0.383	-0.028	0.931	0.019	0.952
BG	Latitude	0.497	0.210	**-0.894**	**0.003**	-0.386	0.215	-0.224	0.484
	MAT	-0.423	0.296	**0.876**	**0.004**	**0.755**	**0.004**	**0.627**	**0.029**
	MAP	**-0.786**	**0.021**	0.452	0.260	0.133	0.679	0.027	0.934
	AI	-0.669	0.069	**-0.904**	**0.002**	**-0.779**	**0.003**	**-0.593**	**0.042**
	T_Sum	0.364	0.375	**0.909**	**0.002**	**0.870**	**0.002**	0.394	0.205
	AI_Sum	0.527	0.179	**-0.848**	**0.008**	-0.394	0.205	-0.387	0.214

Statistically significant correlations are highlighted in bold. MAT: mean annual temperature (°C); MAP: total annual precipitation (mm); AI: aridity index (MAP/PET or potential evapotranspiration). T_Sum (°C) and AI_Sum: averaged mean temperature and aridity indices, respectively, for June, July and August.

**Fig 4:**
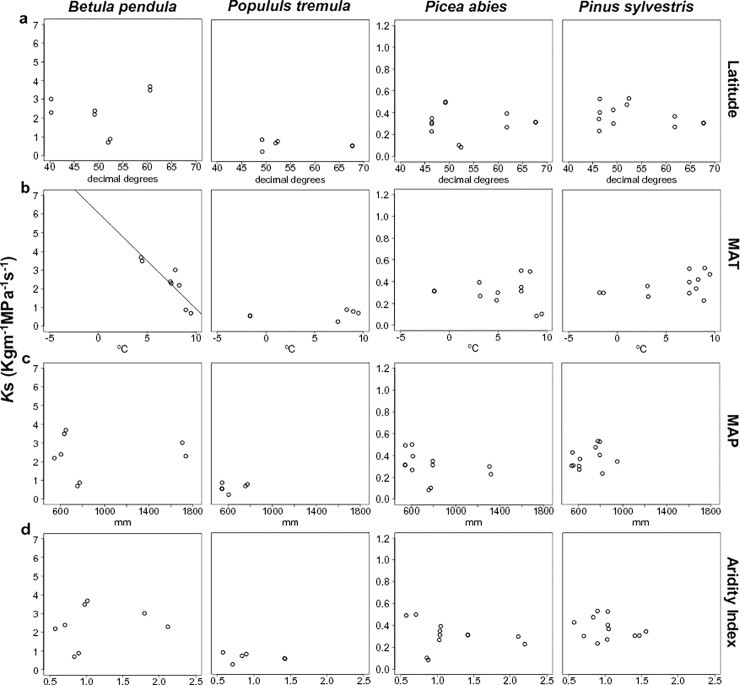
Average xylem-specific hydraulic conductivity (*K*_s_, kg m^-1^ MPa^-1^ s^-1^) per population plotted against latitude (3.a, decimal degrees) and the climatic variables of each sampled population and site: 3.b. mean annual temperature (MAT, °C); 3.c. total annual precipitation; (MAP, mm); 3.d. AI: aridity index (MAP/PET or potential evapotranspiration).

*K*_*s*_ was less strongly related to climate than *P*_*50*_. The *K*_s_/climate correlation was statistically significant only for *Betula pendula*, with lower *K*_s_ values at sites with higher MAT values (Table 3, Fig 4).

**Fig 5:**
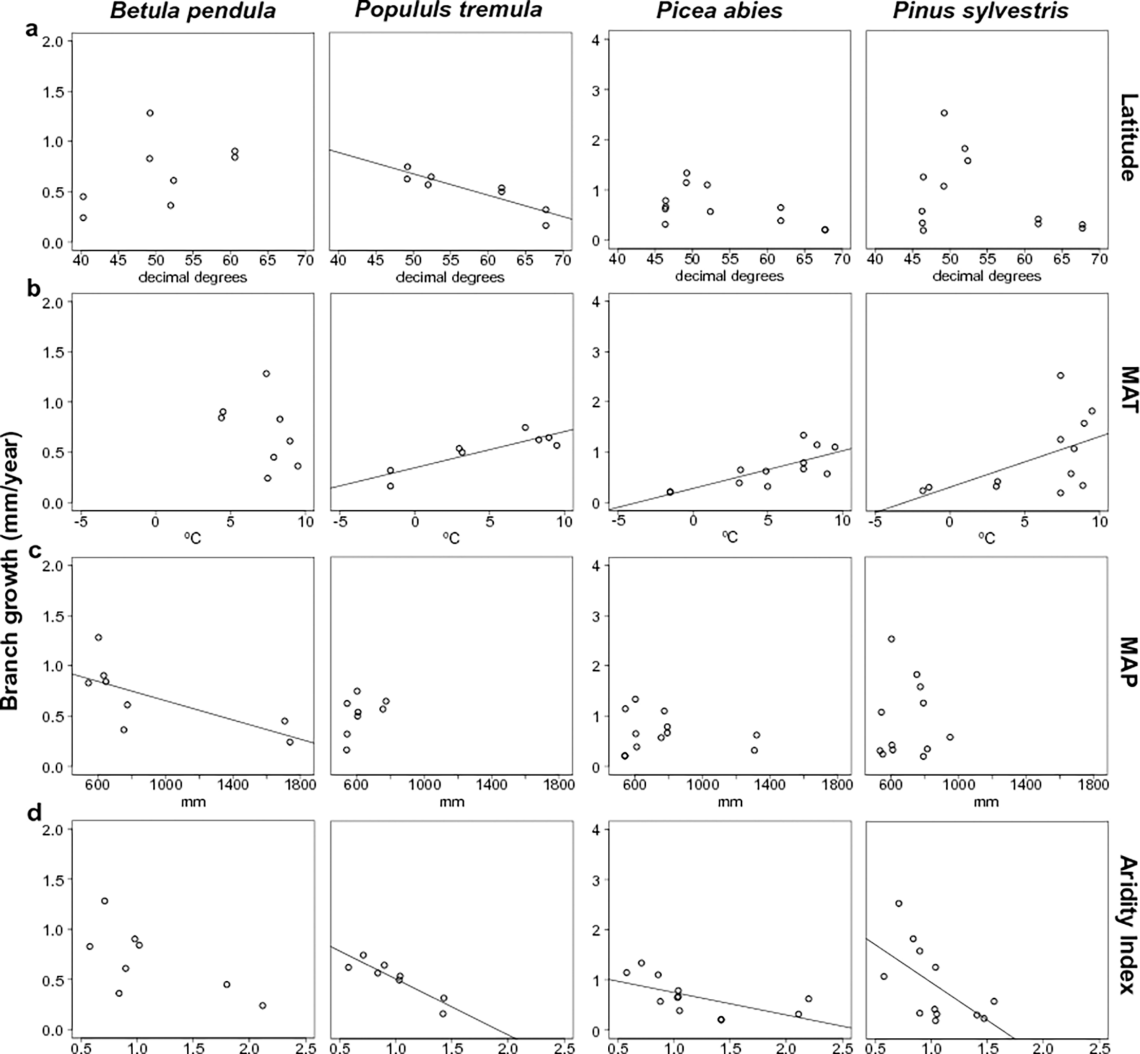
Mean branch growth (estimated as branch radius/xylem age (mm/year)) per population plotted against latitude (5.a, decimal degrees) and the climatic variables of each sampled population and site: 5.b. mean annual temperature (MAT, °C); 5.c. total annual precipitation; (MAP, mm); 5.d. AI: aridity index (MAP/PET or potential evapotranspiration).

We found steeper clines for branch growth than for hydraulic properties, with all species showing at least one statistically significant correlation between branch growth and latitude/climate variables (Table 3, Fig 5). Latitude was correlated with branch growth only in *Populus tremula*, for which the lowest branch growth values were obtained for the northernmost population (Table 3, Fig 5). In general, when statistically significant, branch growth was positively correlated with MAT and T_sum, and negatively correlated with AI, AI_sum and MAP (Table 3, Fig 5).

### Safety-efficiency trade-off

At the intraspecific level, we found statistically significant but weak positive correlations between *P*_*50*_ and *K*_*s*_ for the conifers studied, with the most vulnerable individuals having the largest hydraulic conductivities (Table 4, S3 Fig). No significant correlation was found between *P*_*50*_ and *K*_*s*_ for either of the angiosperms studied (Table 4).

**Table 4:** Correlation coefficients (Pearson or Spearman) for the relationship between *P*_50_ (MPa) and xylem-specific hydraulic conductivity (*K*_s_, kg m^-1^ MPa^-1^ s^-1^) for each tree. Statistical significances are shown.

	*Betula pendula*		*Populus tremula*		*Picea abies*		*Pinus sylvestris*	
	Cor.	*p*	Cor.	*p*	Cor.	*p*	Cor.	*p*
*P*_*50*_ vs *K*_*s*_	0.150	0.274	-0.001	0.995	**0.332**	**0.002**	**0.340**	**0.001**

Statistically significant correlations are shown in bold characters.

## Discussion

We assessed the phenotypic variability of hydraulic safety and efficiency traits (*P*_50_ and *K*_s_, respectively) and branch growth in four tree species across a long latitudinal gradient covering most of their distribution range in Europe. *P*_50_ displayed lower phenotypic variability than *K*_s_ and branch growth, consistent with our initial hypothesis. The low variability of *P*_50_ across populations has been related to uniform evolutionary selection or canalization [37,38]. Indeed, these studies provided evidence of natural selection acting on this trait. This uniform selection reproduces trait conservatism and eventually leads to stasis [37]. In contrast, it has been suggested that *K*_s_ variability is related to the interaction between genotype and environment [35]. *K*_s_ may also vary significantly with sampling position along the branch axis [48,49], although we tried to overcome this limitation through the use of systematic sample preparation procedures. We also expected branch growth to be more variable than *P*_50_, because branch growth is strongly influenced by multiple interacting factors, such as the availability of nutrients, light, water and temperature [46,50,51,52,53] and biotic interactions [54]. The limited embolism resistance variability observed here, in all species other than *Populus tremula*, suggests that forest populations of the studied species will potentially find difficulties to cope with a warmer and drier conditions by increasing their embolism resistance. However, considering the differences in climatic ranges between the studied species, we have to be cautious when interpreting these patterns. Further studies investigating larger precipitation gradients and/or marginal populations are needed. Indeed, a recent study showed that marginal populations of beech significantly differed in embolism resistance [55] while core populations exhibited similar *P*_50_ values [36]. Our results also show that within species phenotypic variability in *K*_s_ and growth are large, and in general larger than that of *P*_50_, suggesting that intra-population variability should not be neglected in further studies at local scales.

Across species, higher resistance to embolism (i.e. more negative *P*_50_ values) have been related to drier environments [26,27]. However, at the intraspecific level, no statistically significant correlations between *P*_50_ and climate have been observed for herbaceous plants [56], angiosperm trees [57, 58] and conifers [34,38,59,60]. Our results are consistent with these findings, as only one species, *Populus tremula*, presented a significant cline in *P*_50_. However, the potential effect of collinearity between climate variables could not be investigated here. Previous studies have reported higher [59,61], similar [34,60] or lower *K*_*s*_ values [35,57] at dry sites than at mesic sites. However, we found no support for a *K*_*s*_/climate cline. In contrast to *P*_*50*_ and *K*_*s*_, all species showed significant correlations between climate and growth. Populations growing at high latitudes and in cold temperatures had the lowest levels of branch growth, probably due to the shorter growing season.

Ideally, plants should be able to maintain both efficient conductivity and the safety of the hydraulic system. However, this is not always the case in natural conditions, and little or no support for a safety-efficiency trade-off has been obtained across species [16]. A few studies have evaluated this trade-off at intraspecific level, and found either no support for the existence of a trade-off [34, 62] or an association of greater conductivities with lower embolism resistance [63]. The lack of correlation between *P*_*50*_ and *K*_*s*_ in angiosperms, and the weak correlation found here for conifers provide insufficient support to conclude that there is a safety-efficiency trade-off. Current knowledge of the anatomical basis of *P*_*50*_ and *K*_*s*_ in conifers is also consistent with the absence of support of such a trade-off, as *P*_50_ is determined principally by the torus-aperture overlap in this clade, whereas *K*_s_ is not related to this anatomical trait [17–19] but rather to the vessel lumen area.

Researchers have recently developed an interest in the phenotypic variability of hydraulic properties [34,35,37,39], due to its possible contribution to community assemblages. However, studies of phenotypic variability can also reveal the potential of the species to adapt to the new environmental conditions imposed by ongoing climate change. We found statistically significant differences in hydraulic safety traits between populations in all the species studied here, and a lack of climate cline in all species other than *Populus tremula*. Overall, this species displayed the greatest variability of *P*_*50*_ within species and between populations, suggesting a potentially greater ability to adapt to environmental changes. This higher variability in safety traits might be due to the fact that *Populus tremula* frequently hybridizes with *Populus alba* in Europe [64]. Yet, the two species significantly differ in numerous phenotypic and ecological properties [65]. In contrast, the low intraspecific variability and lack of climate clines for hydraulic properties in conifers suggest strong genetic constraints, with a much smaller potential to evolve greater embolism resistance in the xylem to cope with the predicted drier conditions. However, hydraulic adjustments can also occur through changes in leaf area: sapwood area ratio [34,66], and a decrease in transpiring leaf area relative to xylem conductive area could hence maintain plant water balance under drought conditions.

## Conclusions

Quantification of the adaptive capacity of populations and species is important for the prediction of natural adaptation to climate change, especially in the long term. Adaptation requires the presence of genetic variation among the individuals of populations upon which natural selection can act. The phenotypic variability in embolism resistance found here was weak and much smaller than that for xylem conductivity and branch growth. In addition, no relationship was found between embolism resistance and climatic variables, except for *Populus tremula*. The species studied (except *Populus tremula*) would therefore be unlikely to be able to adapt hydraulically to drier climatic conditions through the evolution of embolism resistance. Our results provide little support to the existence of a hydraulic safety-efficiency trade-off at the species level.

## Supporting information

S1 Fig Natural distribution areas of the studied species (dark grey) in Western Europe (www.euforgen.org). The triangles represent the populations and sites sampled for this study. CR: Czech Republic; PO: Portugal; NE: The Netherlands; FI-RU: Finland-Ruotsinkylä; FI-VA: Finland-Värriö; FI-HYY: Finland-Hyytiälä; IT: Italy; SW-LOE: Switzerland-Loetschental; SW-PFY: Switzerland- Pfywald.
(DOCX)

S2 Fig Vulnerability curves of each individual and species. Black dot are the raw measure of percentage of loss of conductivity (PLC in %) along the negative pressure gradient (in MPa). The red line connects the PLC fitted by the Pammenter model to the measured xylem pressure. All adjustments were statistically significant.
(DOCX)

S3 Fig *P*_50_ (MPa) versus xylem-specific hydraulic conductivity (*K*_s_, kg m^-1^ MPa^-1^ s^-1^) measured for each tree, for the two species for which *P*_50_/*K*_s_ correlations were statistically significant (*Picea abies* and *Pinus sylvestris*). (DOCX)

S1 Table Species, studied populations and latitude and climate conditions per population. Coordinates are provided in decimal degrees. MAT: annual mean temperature (°C); MAP: annual sum of precipitation (mm); PET: annual sum of potential evapotranspiration (mm); AI: aridity index (calculated as MAP/PET); T_Sum: average temperature of June, July and August (°C); AI_Sum: average AI of June, July and August. Climate data were obtained from WorldClim original 30-s data (http://www.worldclim.org/bioclim) downscaled to 100-m resolution for all but the Italian (IT) and Swiss populations (SW-LOE and SW-PFY), which have their precipitation data from a nearby weather stations at San Vito di Cadore (Centre of Studies of Alpine Environment) and Sierre (www.meteoswiss.ch), respectively, due to highly varying topography. CR: Czech Republic; PO: Portugal; NE: The Netherlands; FI-RU: Finland-Ruotsinkylä; FI-VA: Finland-Värriö; FI-HYY: Finland-Hyytiälä; IT: Italy; SW-LOE: Switzerland-Loetschental; SW-PFY: Switzerland- Pfywald.
(DOCX)
