# Supplementary figures and images for: Quantifying in situ phenotypic variability in the hydraulic properties of four tree species across their distribution range in Europe
Source: PLoS One. 2018 May 1;13(5):e0196075. doi: 10.1371/journal.pone.0196075 (PMC5929519; doi:10.1371/journal.pone.0196075)

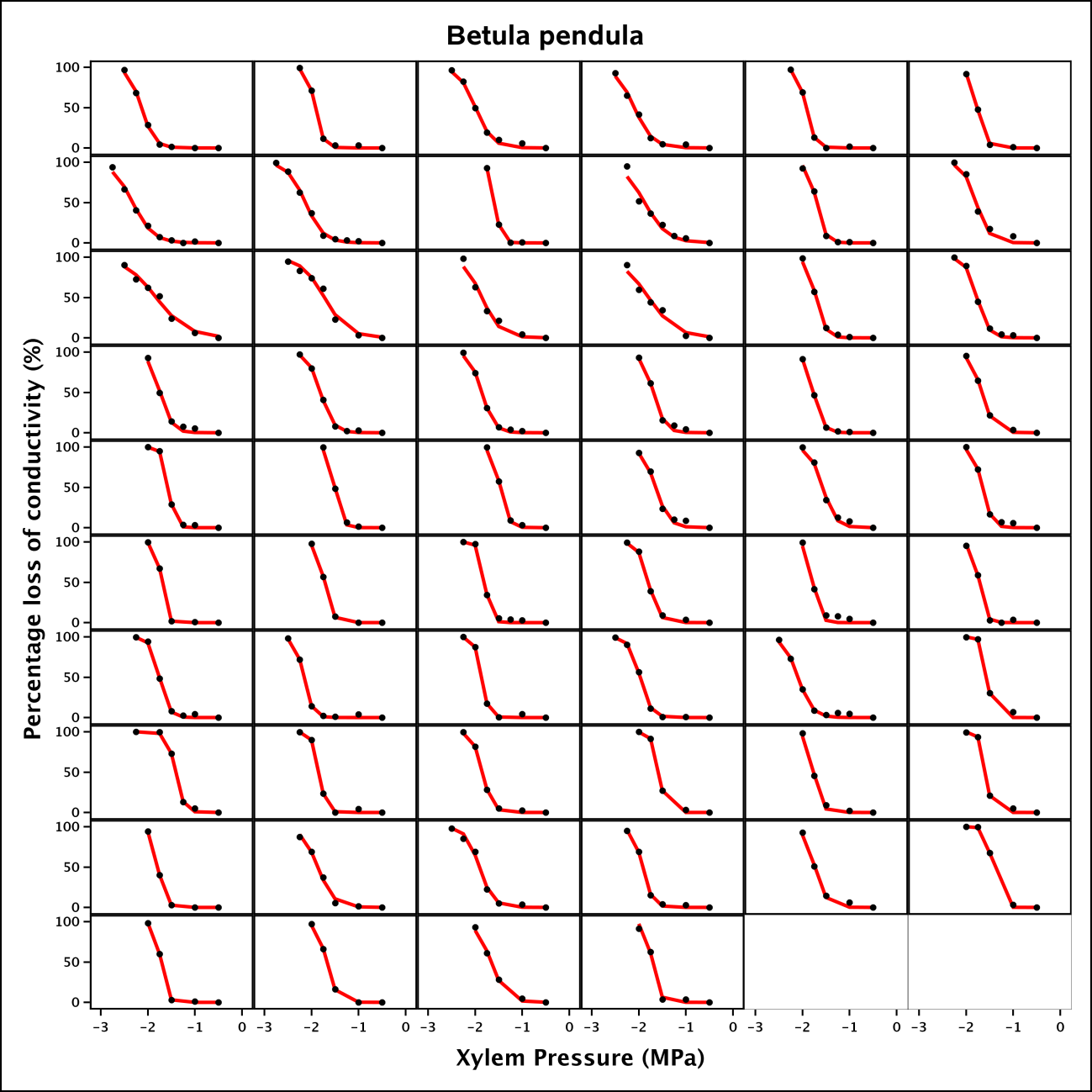


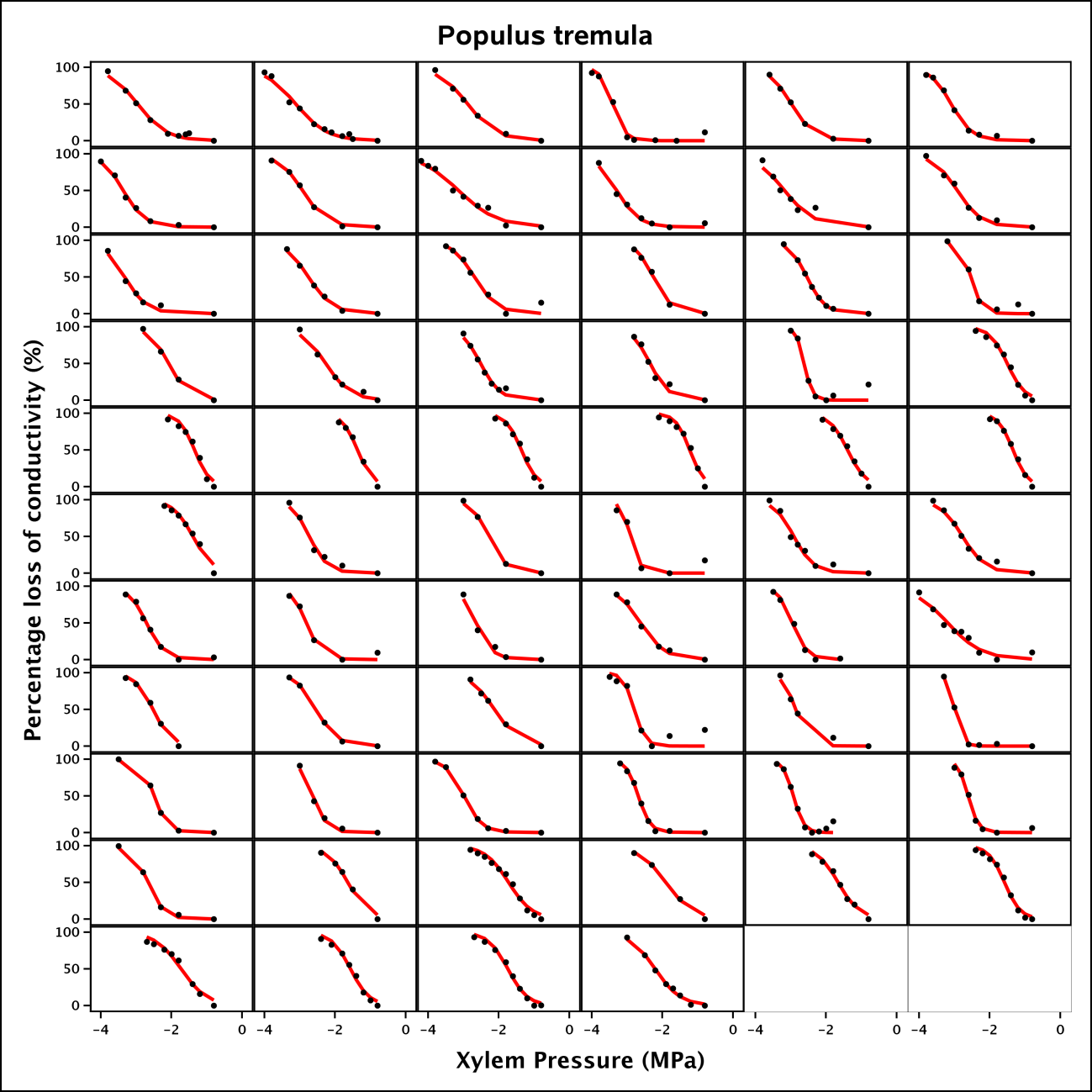


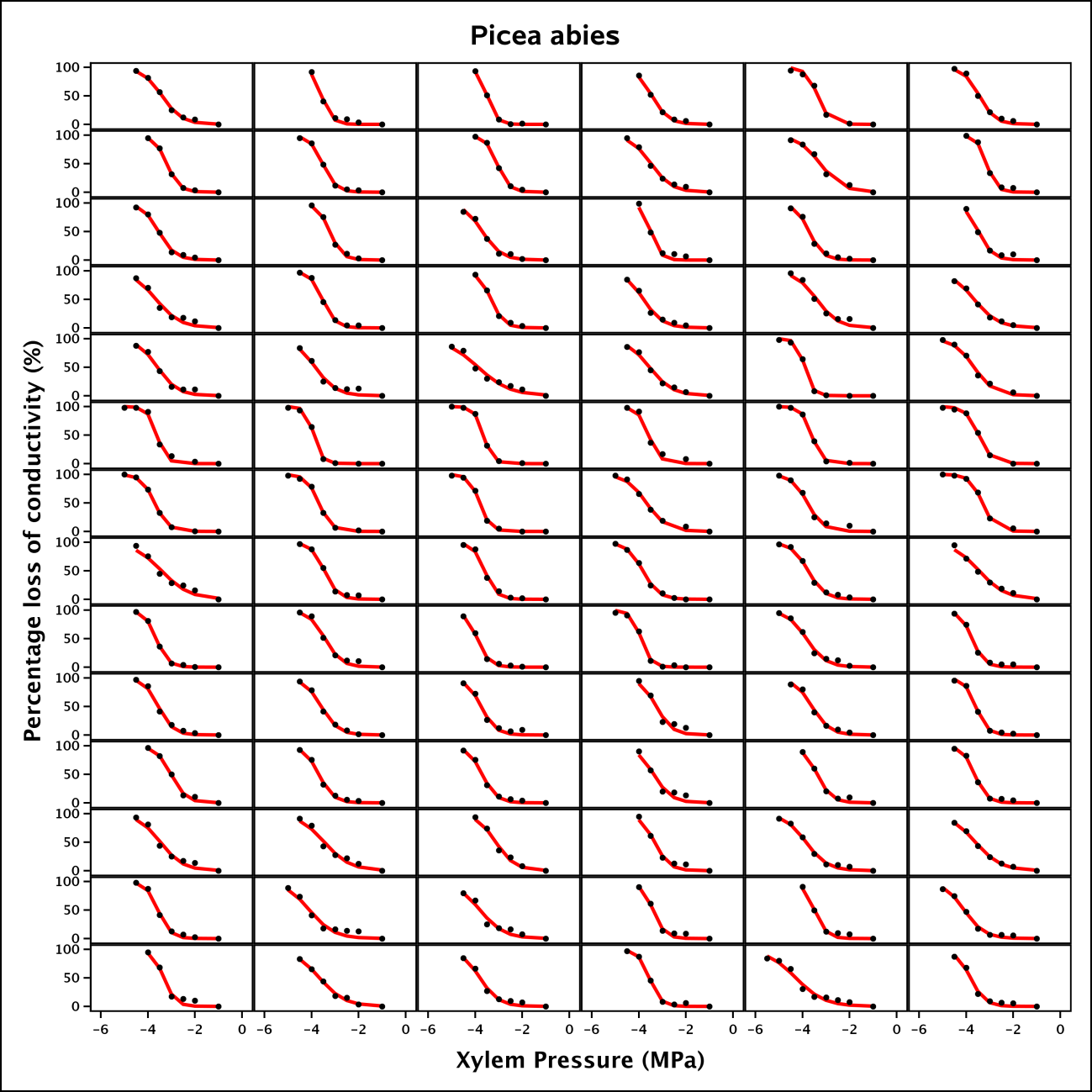


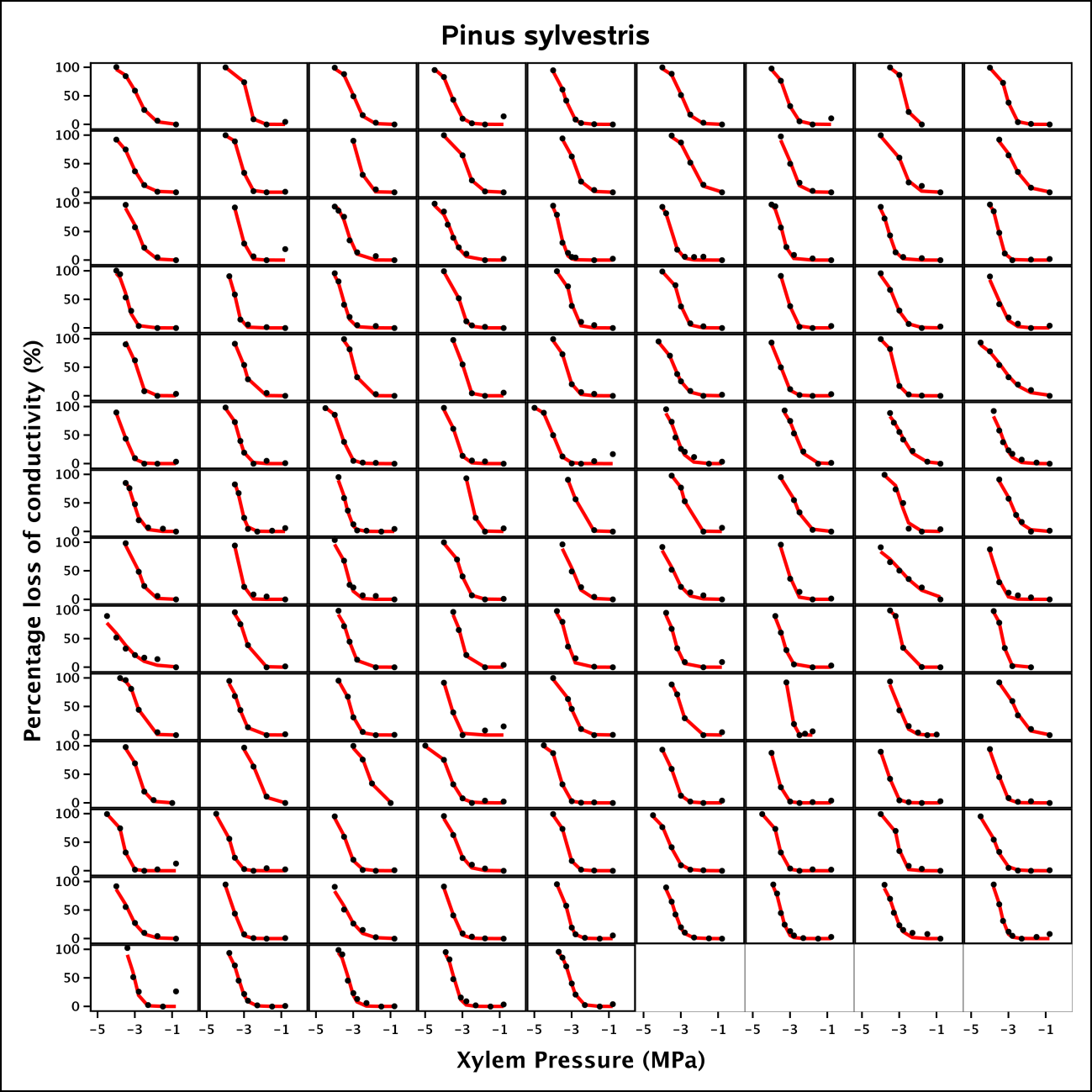

Supplement: S2 Fig — Black dot are the raw measure of percentage of loss of conductivity (PLC in %) along the negative pressure gradient (in MPa). The red line connects the PLC fitted by the Pammenter model to the measured xylem pressure. All adjustments were statistically significant. (DOCX) [file pone.0196075.s002.docx]
