# Supplementary material for: Quantifying in situ phenotypic variability in the hydraulic properties of four tree species across their distribution range in Europe
Source: PLoS One. 2018 May 1;13(5):e0196075. doi: 10.1371/journal.pone.0196075 (PMC5929519; doi:10.1371/journal.pone.0196075)
Supplement: S1 Table — Coordinates are provided in decimal degrees. MAT: annual mean temperature (°C); MAP: annual sum of precipitation (mm); PET: annual sum of potential evapotranspiration (mm); AI: aridity index (calculated as MAP/PET); T_Sum: average temperature of June, July and August (°C); AI_Sum: average AI of June, July and August. Climate data were obtained from WorldClim original 30-s data (http://www.worldclim.org/bioclim) downscaled to 100-m resolution for all but the Italian (IT) and Swiss populations (SW-LOE and SW-PFY), which have their precipitation data from a nearby weather stations at San Vito di Cadore (Centre of Studies of Alpine Environment) and Sierre (www.meteoswiss.ch), respectively, due to highly varying topography. CR: Czech Republic; PO: Portugal; NE: The Netherlands; FI-RU: Finland-Ruotsinkylä; FI-VA: Finland-Värriö; FI-HYY: Finland-Hyytiälä; IT: Italy; SW-LOE: Switzerland-Loetschental; SW-PFY: Switzerland- Pfywald. (DOCX) [file pone.0196075.s004.docx]

| **Species** | **Site** | **Coordinates** | **MAT** | **MAP** | **PET** | **ARI** | **T_Sum** | **ARI_Sum** |
| --- | --- | --- | --- | --- | --- | --- | --- | --- |
|  | PO | 40.32 | 7.7 | 1724.0 | 884.6 | 2.0 | 14.9 | 0.3 |
| *Betula pendula* | CR | 49.25 | 7.8 | 574.5 | 897.9 | 0.9 | 16.2 | 0.5 |
|  | NE | 52.22 | 9.2 | 766.0 | 884.9 | 1.0 | 15.5 | 0.5 |
|  | FI-RU | 60.60 | 4.4 | 642.5 | 643.0 | 0.6 | 16.7 | 0.4 |
|  | CR | 49.25 | 7.8 | 574.5 | 897.9 | 0.6 | 16.7 | 0.5 |
| *Populus tremula* | NE | 52.21 | 9.2 | 767.0 | 884.9 | 0.9 | 16.7 | 0.5 |
|  | FI-HYY | 61.84 | 3.1 | 608.0 | 586.4 | 1.0 | 14.4 | 0.5 |
|  | FI-VA | 67.72 | -1.6 | 544.0 | 381.4 | 1.4 | 10.1 | 0.7 |
|  | SW-LOE | 46.39 | 4.9 | 1316.0 | 610.6 | 2.15 | 12.7 | 1.0 |
|  | IT | 46.43 | 7.4 | 794.0 | 764.8 | 1.05 | 16.3 | 0.8 |
| *Picea abies* | CR | 49.25 | 7.8 | 574.5 | 897.9 | 0.64 | 16.7 | 0.5 |
|  | NE | 52.21 | 9.2 | 767.0 | 884.9 | 0.87 | 16.2 | 0.5 |
|  | FI-HYY | 61.82 | 3.1 | 609.5 | 586.4 | 1.04 | 14.4 | 0.5 |
|  | FI-VA | 67.73 | -1.5 | 543.0 | 382.9 | 1.42 | 10.2 | 0.7 |
|  | SW-PFY | 46.30 | 8.5 | 884.0 | 760.2 | 1.23 | 16.7 | 0.7 |
|  | IT | 46.43 | 7.4 | 794.0 | 764.8 | 1.08 | 16.3 | 0.8 |
| *Pinus sylvestris* | CR | 49.25 | 7.8 | 574.5 | 897.9 | 0.64 | 16.7 | 0.5 |
|  | NE | 52.22 | 9.2 | 766.0 | 884.9 | 0.87 | 16.2 | 0.5 |
|  | FI-HYY | 61.82 | 3.1 | 609.5 | 586.4 | 1.04 | 14.4 | 0.5 |
|  | FI-VA | 67.74 | -1.6 | 545.5 | 379.8 | 1.44 | 10.1 | 0.7 |
