# Supplementary material for: Quantifying in situ phenotypic variability in the hydraulic properties of four tree species across their distribution range in Europe
Source: PLoS One. 2018 May 1;13(5):e0196075. doi: 10.1371/journal.pone.0196075 (PMC5929519; doi:10.1371/journal.pone.0196075)
Supplement: Supplementary file 3 [file pone.0196075.s001.docx]

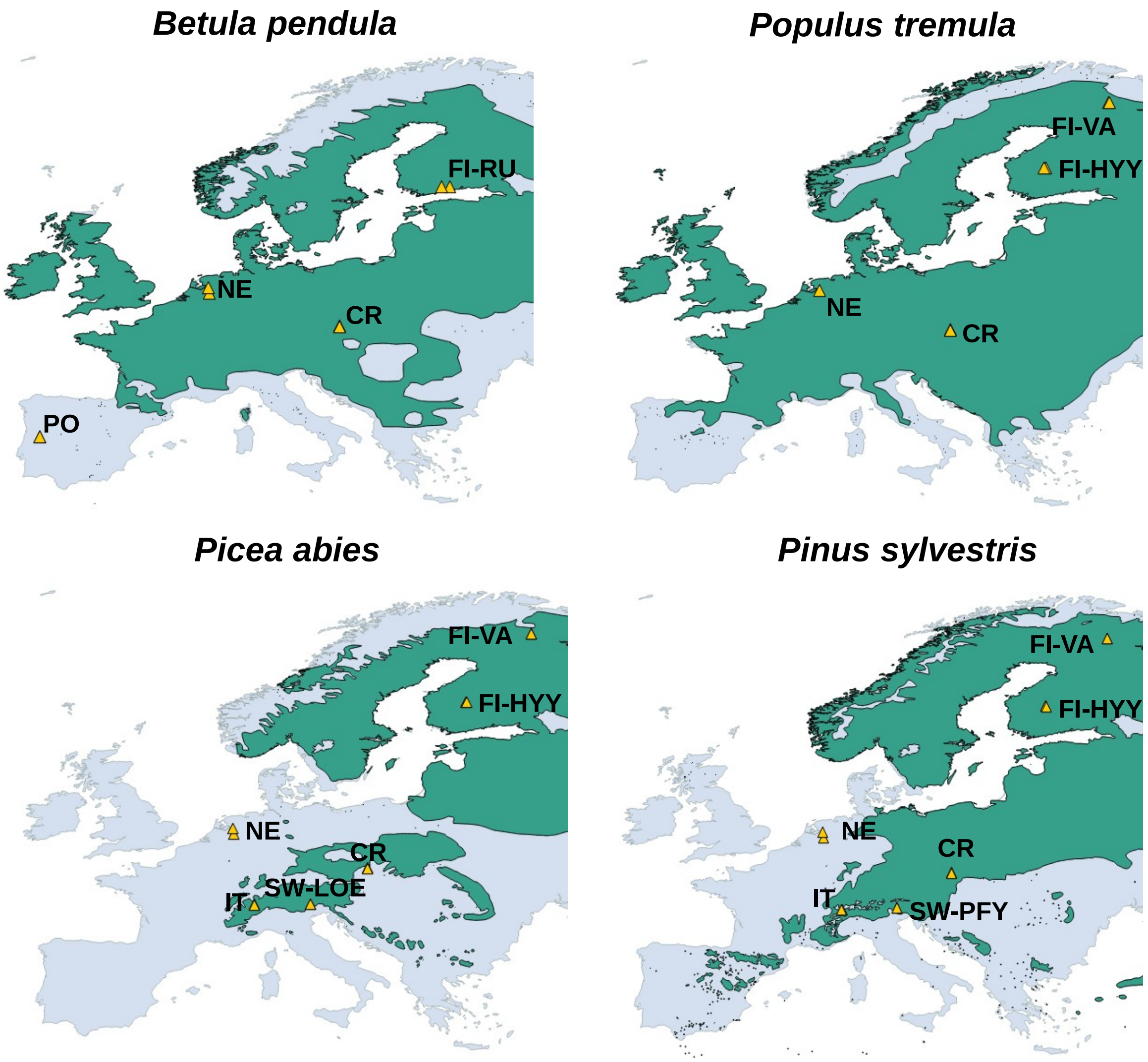
Betula pendula
FI-RU
NE
CR
PO
Populus tremula
FI-VA
FI-HYY
NE
CR
Picea abies
FI-VA
FI-HYY
NE
CR
IT
SW-LOE
Pinus sylvestris
FI-VA
FI-HYY
NE
CR
IT
SW-PFY
