# Supplementary material for: Quantifying in situ phenotypic variability in the hydraulic properties of four tree species across their distribution range in Europe
Source: PLoS One. 2018 May 1;13(5):e0196075. doi: 10.1371/journal.pone.0196075 (PMC5929519; doi:10.1371/journal.pone.0196075)
Supplement: Supplementary file 4 [file pone.0196075.s003.docx]

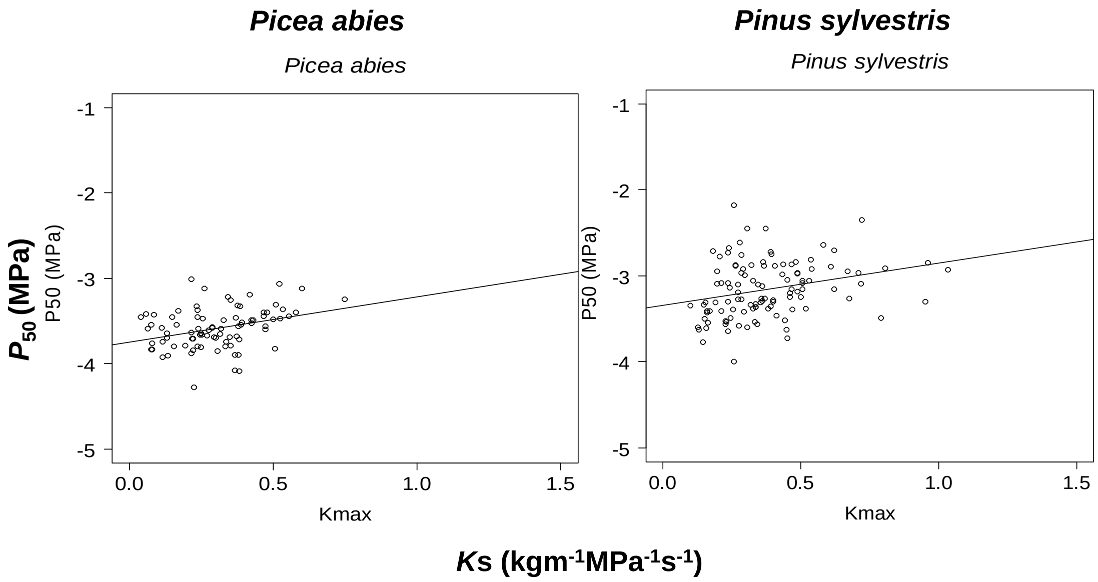

Picea abies
Picea abies
Pinus sylvestris
Pinus sylvestris
P50 (MPa)
P50 (MPa)
P50 (MPa)
-1
-2
-3
-4
-5
0.0
0.5
1.0
1.5
Kmax
Kmax
Ks (kgm-1MPa-1s-1)
